# Challenges Experienced by Italian Nursing Home Staff in End-of-Life Conversations with Family Caregivers during COVID-19 Pandemic: A Qualitative Descriptive Study

**DOI:** 10.3390/ijerph19052504

**Published:** 2022-02-22

**Authors:** Silvia Gonella, Paola Di Giulio, Alexandra Antal, Nicola Cornally, Peter Martin, Sara Campagna, Valerio Dimonte

**Affiliations:** 1 Department of Public Health and Pediatrics, University of Torino, Via Santena 5 bis, 10126 Turin, Italy; silvia.gonella@unito.it (S.G.); alexandra.antal.90@gmail.com (A.A.); sara.campagna@unito.it (S.C.); valerio.dimonte@unito.it (V.D.); 2 Direction of Health Professions, Azienda Ospedaliero Universitaria Città della Salute e della Scienza di Torino, Corso Bramante 88–90, 10126 Turin, Italy; 3 Catherine McAuley School of Nursing and Midwifery, Brookfield Health Sciences Complex, University College Cork, College Road Cork, T12 AK54 Cork, Ireland; n.cornally@ucc.ie; 4 School of Medicine, Faculty of Health, Deakin University, 75 Pigdons Road, Waurn Ponds, VIC 3216, Australia; peter.martin@deakin.edu.au

**Keywords:** challenges, communication, conversation, COVID-19, education/training, end of life, family caregivers, healthcare professionals, nursing homes, qualitative research

## Abstract

End-of-life conversations are among the most challenging of all communication scenarios and on the agenda of several healthcare settings, including nursing homes (NHs). They may be also difficult for experienced healthcare professionals (HCPs). This study explores the difficulties experienced by Italian NH staff in end-of-life conversations with family caregivers (FCs) during COVID-19 pandemic to uncover their educational needs. A qualitative descriptive study based on inductive thematic analysis was performed. Twenty-one HCPs across six Italian NHs were interviewed. Four themes described their experiences of end-of-life conversations: (1) communicating with FCs over the overall disease trajectory; (2) managing challenging emotions and situations; (3) establishing a partnership between HCPs and FCs; (4) addressing HCPs’ communication skills needs. HCPs had to face multiple challenging situations that varied across the care period as well as complex emotions such as anxiety, guilt, uncertainty, fear, anger, or suffering, which required tailored answers. COVID-19 pandemic increased FCs’ aggressive behaviors, their distrust, and uncertainty due to visitation restrictions. HCPs had to overcome this by developing a set of strategies, including adoption of an active-listening approach, supportive communication, and explicit acknowledgement of FCs’ emotions. Since communication needs were mostly practical in nature, HCPs valued practical communication training.

## 1. Introduction

End-of-life conversations are some of the most challenging of all communication contexts and occur in a variety of healthcare settings, including nursing homes (NHs) [1,2]. These conversations may involve patients and/or their family caregivers (FCs) and cover discussions about clinical conditions, course of disease and prognosis, treatment goals and options, symptom management, end-of-life wishes, and plans for the future care [3]. End-of-life conversations comprise at least four processes: information gathering, information sharing, responding to emotions, and fostering relationships [4]. When these elements are done skillfully, end-of-life conversations promote trust and therapeutic alliance between healthcare professionals (HCPs) and patients and their FCs [5]. Instead, ineffective communication can result in suboptimal care, undue psycho-emotional distress, and FCs’ dissatisfaction with the care received [5,6]. Having end-of-life conversations with patients or at least their FCs to elicit preferences for care at the end of life is essential to align care with patients’ goals and values [7]. Among seriously ill older persons such as NH residents, this goal-concordant end-of-life care is one of the top-ranked quality indicators for palliative care throughout the overall disease trajectory [7,8].

Despite the consensus that end-of-life conversations should be initiated at the earliest possible stage in a life-limiting illness to plan future care and treatment in advance [9], and that the literature supports collaborative models in which end-of-life conversations require a team [10], it is often left to individual institutions and their HCPs to decide when and how these conversations are conducted and by whom [11]. Thus, it is not surprising that end-of-life conversations are often postponed due to their challenging nature until the patient’s condition acutely deteriorates, when recovery is no longer possible [12]. This delay in offering end-of-life conversations has been often justified by prognostication difficulties in patients with multimorbidity [13].

FCs usually expect HCPs to start end-of-life conversations [12,14], and HCPs acknowledge they should take a leadership role in this regard; conversely, they are uncertain about the right time to initiate such conversations [15,16]. The timing is often determined by the patient’s health status, and HCPs usually wait for physical (e.g., weight loss, swallowing difficulties) and/or social (e.g., level of readiness to initiate an end-of-life conversation) cues to prompt these conversations [17,18]. Cues from patients and FCs as well as HCPs’ intuition that they are open to such conversations also play an essential prompting role [15]. Several follow-up conversations that break information up into multiple chunks may be needed to promote patients’ and FCs’ understanding and finally, shared decision-making [17]. HCPs should give patients and FCs opportunities to engage in end-of-life conversations whilst being sensitive to their communication needs, preferences, and state of readiness for such dialogue [19]. Therefore, deferring end-of-life conversations when patients or FCs are not comfortable and raising them again at a later time may be required, thus requiring HCPs’ continuous engagement. This suggests that HCPs have to both initiate and refine end-of-life conversations. Moreover, most HCPs feel underprepared and lack competence/confidence in end-of life conversations due to limited training of how to communicate with seriously ill patients and their FCs [13,16,20]. Some HCPs report they develop communication skills in the field, experience discomfort with planned formal conversations, and prefer spontaneous conversations, thus denoting a condition of vulnerability [15].

In addition to these well-known barriers, the COVID-19 pandemic has forced further changes in both the content and modality of end-of-life conversations. The International Association for Communication in Healthcare and the Academy of Communication in Healthcare recognized that with COVID-19, further bad news may include that FCs cannot see their relatives for a period of time because they are in quarantine, or a loved one’s worsened conditions or death due to COVID-19 disease [21]. HCPs had to establish meaningful conversations in a virtual environment by employing information and communication technologies (ICTs), and lack of face-to-face contact prevented them from relying on nonverbal communication strategies [22]. Qualitative research highlighted that HCPs felt it was challenging to have sensitive communication about prognosis and provide psychological and social support to FCs over the phone, particularly when no previous rapport had been established [23]. This exposes HCPs to overwhelming stress, anxiety, depression, insomnia, burnout, and difficulties managing prognostic uncertainty [21,24]. Therefore, targeted interventions and resources to improve resilience and communication skills among frontline HCPs would be beneficial [21,24].

Despite considerable attention being paid to the impact of COVID-19 on NH settings, public scrutiny has been mostly given to the prevention and control of infection spreading and the impact of visitation restrictions on residents’ and their FCs’ well-being [25], while less attention has been paid to challenges experienced by HCPs in daily practice, particularly in actualizing the recommendations to strengthen NH–FCs communication channels [26]. We conducted a qualitative study to explore the challenges experienced by Italian NH staff in both initiating and continuing end-of-life conversations with FCs during COVID-19 pandemic, to gain knowledge on what type of communication skills training to prioritize for HCPs who work in NHs.

## 2. Materials and Methods

### 2.1. Research Design

A qualitative descriptive study was performed from May 2021 to August 2021. The qualitative descriptive study provides a comprehensive summary of events in their everyday terms and is the method of choice when straight description of phenomena is desired. Researchers stay close to the data and to the surface of words and events [27]. To ensure methodological rigor in reporting, the Consolidated Criteria for Reporting Qualitative Studies (COREQ) guidelines were followed [28].

### 2.2. Setting and Recruitment

Forty-four Northwest Italian NHs were purposively approached for their geographical area to guarantee the greatest variation of data, and six NH managers accepted to participate on a voluntarily basis. Characteristics of participating NHs are reported in Table 1.

NH managers were preliminarily approached by telephone and received the study protocol by email. NH managers purposively identified HCPs (a) of any profile, (b) with at least 6 months experience in the facility, (c) responsible for the communication with FCs in any phase of care (i.e., from admission to end of life), and (d) willing to participate in the study. Administrative staff were excluded as their role does not involve end-of-life conversations with FCs.

Eligible HCPs were contacted by phone by the research team after preliminary contact by the NH manager.

It was desirable to recruit at least 12 HCPs of the NH workforce because elements for themes arise within 12 interviews [29].

### 2.3. Participants

A total of 21 HCPs (four NH managers, four chief nurses, three chief medical officers, three nurses, three psychologists, two educators, one chief nurse aide, and one nurse aide) participated in the study.

Most HCPs were female (*n* = 17), and the mean age was 50 years (range: 25–73) (Table 2).

All participants had face-to-face interviews in a quiet and private room of the facility before, at the end of, or during their working shift. No one other than the participants and the researcher was present at the interview to open up for possible negative comments and allow the participants to speak without interruption. The mean duration of interviews was 37 min (range: 21–67).

### 2.4. Data Collection

**Figure 1:**
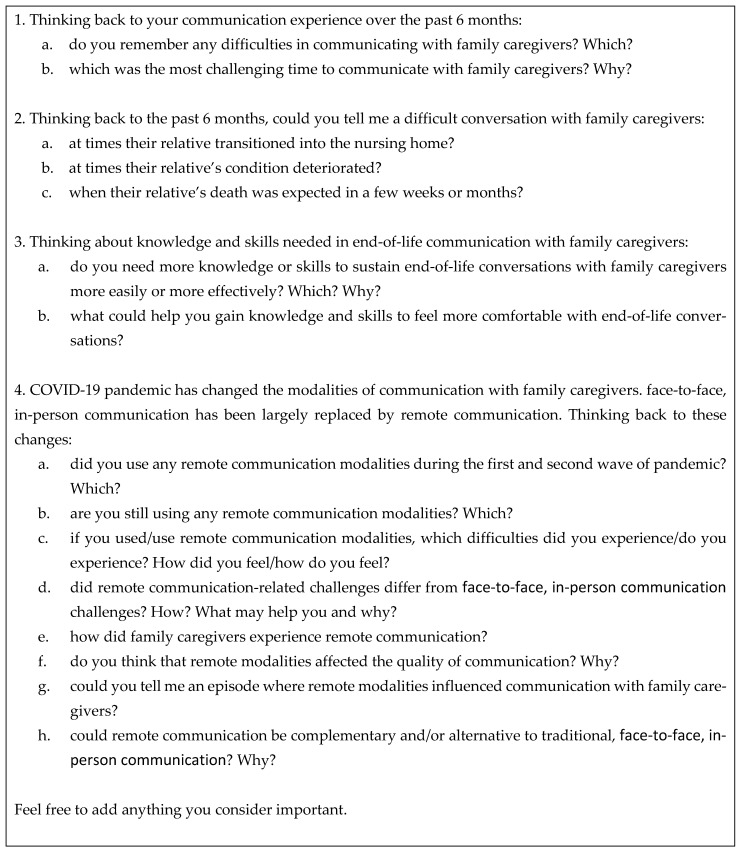
Interview guide.

A researcher (A.A.) not affiliated to the NH conducted semistructured, in-depth interviews and employed probes to stimulate participants’ narratives. The interview guide was based on relevant literature [30,31] and the experience of three experts (P.D.G., S.G., and V.D.) in qualitative methodology and end-of-life care. The semistructured interview guide (Figure 1) was refined after the first two interviews and explored (1) HCPs’ experience of end-of-life communication with FCs during the pandemic; (2) challenges experienced by HCPs in initiating or continuing end-of-life communication when residents are admitted to a NH, when a resident’s condition deteriorates, and in the last weeks or few months of a resident’s life (hereafter end of life); (3) HCPs’ communication skills training needs, and (4) HCPs’ perspective about ICT-based communication with FCs. During the interview, in-the-field notes of nonverbal communication were recorded in a reflective diary.

Additional data were collected about (i) HCPs’ profile (i.e., gender, age, education, professional profile, overall working experience and experience in NHs, and employment), and (ii) NHs’ profile and their working processes. The NH managers questionnaire explored structure variables (e.g., public or private NH, staffing), process variables (e.g., written procedures, meetings with FCs and residents, documentation of end-of-life care preferences), and outcome variables (referral of residents to palliative care services in the 6 months before study start, extra activities such as pet or music therapy).

### 2.5. Transcription and Qualitative Data Analysis

A.A. transcribed the interviews verbatim immediately after the interview, and S.G. randomly checked 10 transcripts for accuracy. Participants could review their transcripts; however, none requested copies. Data collection and analysis were conducted simultaneously to inform the subsequent interviews. Inductive thematic analysis that relies on inductive reasoning with categories and themes emerging from the raw data through repeated examination and comparison was performed [32]. Two researchers independently analyzed interview transcripts using the software ATLAS.ti 8. They met after each set of three interviews to discuss developing coding sheets and resolve discrepancies. All transcripts were reread as new codes were developed. The final coding sheet was shared among all research team members, who gathered similar codes in categories, and similar categories in themes. Themes are illustrated by participants’ quotations, which are identified by a code indicating the NH and the HCPs’ profile (e.g., NH1/nurse, NH1/psychologist, NH2/NH manager).

### 2.6. Rigor and Trustworthiness

Guidelines for trustworthiness were followed [33]. To ensure credibility and dependability, the two coders met after each set of three interviews to consolidate codes, thus increasing reflexivity. Moreover, all team members reviewed the coding process and agreed on categories and themes. Confirmability was pursued through quality checking of interview transcripts and triangulation within the team to identify categories, themes, and significant excerpts, as well as by keeping an audit trail over the entire study to clarify the data collection and analysis. Transferability was enhanced by describing the data collection process and sample characteristics and seeking data saturation. Finally, authenticity was ensured by establishing a trusting relationship with the interviewees and putting them at ease in a quiet setting without external interferences [34].

### 2.7. Quantitative Data Analysis

Quantitative demographic data and NH-related variables were computed as frequencies, percentages, mean with standard deviation, or range.

### 2.8. Ethical Considerations

The study protocol was approved by the Ethics Committee of the University of Torino. All participants provided written informed consent to participate in the study and be audio-recorded after information about the study objectives and data collection procedure was provided. Participants could discontinue the interview at any time and for any reasons. Transcripts were anonymized for both the NH and the participant.

## 3. Results

HCPs’ experience of end-of-life conversations with FCs of NH residents during COVID-19 pandemic was summarized in 23 categories, which were gathered in four themes (Table 3; Table S1).

### 3.1. Theme 1. Communicating with Family Caregivers over the Overall Disease Trajectory

All HCPs highlighted the essential role of a supportive communication over the entire care period. Communication needs to be clear, truthful, continuous, and provide a source of emotional support for FCs. Moreover, communication should be tailored to each FC by considering their specific information needs in terms of both frequency and content, their awareness about a relative’s clinical condition, cultural background, and emotional state at the time of the meeting. To be really supportive, communication requires professional competence and caring attributes such as empathy, kindness, sensitivity, humanity, and patience, in addition to skills including the ability to actively listen, suspend judgment, and remember that silence may be highly valued in these conversations; it should be provided by HCPs who are well-informed and have full understanding of the situation.

*“The most important skill is adjusting to the person in front of you […], my role requires me to be supportive and welcoming.”*

(NH4/NH manager)

*“Professionalism is having a full understanding of the situation, not only what happens during your shift […]. Thus, if they [FCs] ask me for advice you can provide the right guidance.”*

(NH5/nurse)

*“We funnel communication into a contact person to avoid saying things that should not be said or are incorrect. When you give wrong information, you create fear. Therefore, communication should be centralized on specific figures who are responsible for interacting with and updating FCs.”*

(NH6/chief medical officer)

All HCPs preferred in-person communication over ICT-based communication regardless of the phase of illness, if possible. In-person communication allowed HCPs to capture FCs’ nonverbal behaviors, support them through body language, and foster relationships based on trust. ICT-based communication (e.g., by telephone, text messages, e-mails, video calls) was perceived as complementary to in-person communication and useful in specific situations, including urgent communications or when the presence of FCs in the NH was not allowed or difficult, such as with visitation restrictions in place or when FCs live far from the NH or suffer from mobility-limiting health problems. Interviewees reported that ICT-based communication has several limitations: misunderstandings may be more frequent as well as the risk for “missing pieces”, and rapport is threatened by the lack of body language and interpretation of understanding.

Communication with FCs was perceived as an ongoing process tailored to a resident’s disease progression and consequently to changing FCs’ needs. The care during the stay at the NH was a period of mutual acquaintance, and HCPs had to collect information about a resident’s home routine to provide the best person-centered care. This phase was often challenging for FCs, and HCPs had to sustain them in the decision to admit their relative to a NH.

*“Sometimes they [FCs] feel really guilty for leaving their relative here and not being close to them, and therefore we must reassure them, they need this.”*

(NH3/nurse)

When a resident’s condition deteriorated, HCPs had to provide FCs with information about life expectancy, the possibility of impending death, and the dying process. HCPs perceived this communication as one of the most emotionally challenging and reported greater difficulties when they had to introduce such conversation for the first time, especially when unexpected. Instead, a strong rapport made this much easier. During COVID-19 pandemic, HCPs increased the frequency of updates after acknowledging clinical deterioration to support and stay close to FCs.

*“For me, to call and say, ‘Conditions are deteriorating’ or ‘Something is going wrong’, that’s the hardest time. I’d prefer that other colleagues do this communication”*

(NH4/NH manager)

Contact with FCs did not end with death and continued throughout the acute bereavement phase. When sincere and mutual respectful relationships had been established during the stay at the NH, HCPs perceived FCs to be satisfied with the communication received and the resident’s death was experienced as a journey coming to end. This difficult phase was even more challenging when FCs could not be present at the bedside, as during COVID-19 pandemic.

*“There’s a feeling of accomplishment, it’s not possible to go back so you really have to make the effort to get through that moment and give them [FCs] what they need. I think that’s the hardest part of the journey for me.”*

(NH2/chief medical officer)

*“Before COVID it was easier, now it is less. Before we said ‘Come to the facility, let’s have a meeting all together with the head nurse, the physician and the chief medical officer and let’s try to make a picture’. Now it is more difficult to organize these meetings and it has become harder to make FCs accept death.”*

(NH5/NH manager)

### 3.2. Theme 2. Managing Challenging Emotions and Situations

HCPs reported several challenging situations they had to tackle, including FCs’ denial of a resident’s clinical deterioration or prognostic discordance. Particularly, FCs of persons with dementia had difficulties accepting dementia as a cause of death. When FCs were unaware about their relative’s health condition, they doubted the information received from HCPs and were more likely to be surprised as the end of life approached or ask for interventions that HCPs judged disproportionate for the resident, such as oral feeding in presence of swallowing difficulties. The pandemic further hindered FCs’ perception of their loved one’s health because of visitation restriction, which prevented them from directly observing changes.

*“We have great difficulties with relatives of persons with dementia. They often have not realized yet the situation of their loved one, that there is a cognitive decay and disease progresses even if we are doing everything we can. Some people do not want to understand and communication becomes extremely difficult.”*

(NH6/psychologist)

HCPs often had to manage FCs’ mismatched expectations, which needed to be addressed:

*“The daughter said ‘I was expecting a phone call every day’ and I calmy replied ‘It’s not possible call every day’. Then we shared that usually we don’t call FCs if no issues occur; we call FCs when something is going wrong or changing”.*

(NH6/chief nurse)

*“The expectations of FCs are very high, so especially at the end of the meeting I underline the limits of the structure and difficulties that are likely to arise to prevent unfulfilled expectations from leading to aggressive behaviors”.*

(NH2/NH manager)

Moreover, HCPs had to manage their own uncertainty about prognosis and complex FCs’ emotions from admission to death. During the admission phase, they were confronted with FCs’ feelings of anxiety, uncertainty, and guilt, and their fear of a relative deteriorating after admission due to changed routine. Then, approaching and at the end of life, they had to contain anger, aggression, distress, and grief. Moreover, interviewees reported increased aggression, dissatisfaction, and distrust from FCs during the pandemic, since entering the facility was not allowed, in addition to fear for their relative dying alone.

*“Almost everyone asks: ‘How much time is left?’ And you don’t know what to answer because they [residents] often get worse, then stabilize, and sometimes they even seem to recover a little”.*

(NH2, head nurse)

*“During pandemic it has been really hard. In usual conditions as it was some years ago, FCs could be angry but then they could enter the facility and see, you could explain and show them all that you had done and all that was still continuing to be done. Finally, they took confidence and also changed their behaviors. All of this was no more possible during COVID.”*

(NH6/chief nurse)

Finally, caring for persons at the end of life required HCPs to manage their own challenging emotions. Some HCPs reported reliving painful personal experiences. During the pandemic, they suffered seeing residents dying without their relatives at the bedside or witnessing ICT-based communication between FCs and their actively dying relative. Moreover, they were fearful of FCs’ emotional reactions after bad news.

*“When you have elderly parents as I have, you really feel involved. That resident may be you mum or dad … at that moment there is a very strong transfer in reverse. You really live it in a heavy way, you can’t detach anymore.”*

(NH2/psychologist)

*“Some children wanted to see their relatives even though they were almost in coma. It was very challenging for me to make this video call and mediate between children and the dying resident”.*

(R5/educator)

Interviewees identified some strategies they adopted to overcome challenges, including explicitly acknowledging FCs’ emotions to provide tailored answers, helping FCs to acknowledge their relative’s changed clinical condition, adopting an active-listening approach, focusing on the content of communication, and transferring responsibility of communication to the physician, the NH manager, or the chief medical officer.

*“The FC often does not have a real understanding of what’s going on […]. It’s actually a lot of little signals that come through, so we need to say ‘Your mum/father needs some help now to eat’. I mean, we need to point out all the little signals that occur. We need to accompany FCs towards a new vision of their relative and this is a hard transition.”*

(NH2/psychologist)

Moreover, HCPs relied on multi-professional meetings and reported that it would be helpful to define which information be given based on the professional role. Finally, the importance of also training administrative staff in engaging with FCs has been highlighted.

*“Everyone should recognize their role: which information could I provide if I’m asked? Only that concerning my role. This stuff creates so many problems. All professionals should be aware of their limits; I can answer up to a certain point, then I refer to the responsible person […] Thus, boundaries among roles are recognized. This is pivotal to provide right communication, what you say is correct within your scope, you don’t create confusion.”*

(NH5/NH manager)

*“Administrative staff do not have to explain anything to FCs since they do not know anything but are intermediary, they have to learn how to mediate […]. This position is important if we want to establish good relationships with FCs.”*

(NH3/chief medical officer)

### 3.3. Theme 3. Establishing a Partnership between Healthcare Professionals and Family Caregivers

Most HCPs stated that it is critical to understand the needs of FCs and personalize how to support them. Some FCs needed detailed clinical information, others reassurance and emotional support, and to stay in touch with their relative regardless of the modality (e.g., in-person visits, window visits, outdoor visits, video calls).

*“Some FCs desire updating about the clinical health status and everything else is secondary. Others want to be informed about daily routine, if the mom has eaten, slept … and still others want to be reassured that their relative does not feel abandoned. We have to avoid mixing the plans and provide FCs what they need.”*

(NH3/chief medical officer)

All HCPs agreed that establishing relationships based on trust with FCs required commitment. Such relationships were facilitated by matched care goals between HCPs and FCs, and promoted by in-person communication, while they were hindered when FCs were intrusive or critical, or if internal conflicts occurred in the family unit.

Most HCPs stated that trusting relationships were built over time and pivotal for true shared decision-making about goals of care at the end of life, including the decision to hospitalize and the preferred place of death. Some HCPs reported difficulties supporting FCs when they desired to pursue curative-oriented care when comfort-oriented care had been suggested. In other cases, disagreement about the intensity of care arose among HCPs.

*“The relationship built with FCs over years is essential. If you establish good relationships, then when you get to the epilogue, death is peaceful with no aftermath. Short stays are more difficult because you haven’t had the time to build any relationships.”*

(NH2/NH manager)

*“It happens that you have to fight with the doctors because they say, ‘She had a seizure, now we’ll bring her to the hospital to do a CT scan to figure out …’. ‘No’ I reply ‘once we’ve done the CT scan, the MRI and all the examinations, what do we do? Will she have surgery? No…’. So, we care for her humanely. To hospitalize means leaving them dying alone.”*

(NH1/NH manager)

HCPs often struggled to explore FCs’ preferences for care at the end of life due to the lack of well-developed advance care directives. Most FCs still perceived death as a taboo topic and were moved by a filial duty to leave nothing undone, and frequently believed they would be abandoning their relative if a comfort-oriented approach was chosen. The interviewees often indirectly explored FCs’ preferences for the desired care at the end of life due to their sensitivity to FCs’ feelings. However, they also reported that FCs’ care preferences may change and usually move towards a palliative-oriented approach when the resident’s clinical condition deteriorates.

*“Initially they [FCs] say ‘Let’s do it, let’s do all what we can, let’s go to the hospital”, then as years pass, they [FCs] acknowledge disease-related suffering and tell you ‘If possible we would like (s)he stays here among people (s)he knows and we are able to stay with her/him as well”.*

(NH4/NH manager)

HCPs also reported several obstacles to a partnership with FCs, including (1) fragmented communication with and within the care team (i.e., FCs received conflicting information from different HCPs and across services), (2) high turnover, (3) staff shortages, which had further worsened during the pandemic with several HCPs moving to the hospital, and (4) lack of time due to high workload, particularly during the first wave of pandemic when residents needed high intensity of care.

*“Time really lacks, time is tyrant. If we had the opportunity to have more time, we would speak more with FCs and achieve solutions faster. When there is communication you are halfway there.”*

(NH6/chief nurse)

### 3.4. Theme 4. Addressing Healthcare Professionals’ Communication Skills and Training Needs

Most HCPs believed that communication skills are innate, even if they can also be learnt through experience and training programs. Some interviewees reported to have gained communication skills by attending training programs during their education or while caring for a relative. However, most stated that working experience was the best teacher. Several HCPs would be interested in attending communication training programs, but education initiatives offered by NHs were poor or absent.

*“I communicate well with FCs because I had the same experience with my parents, thereby I understand how they feel and know what they want to hear, what they need.”*

(NH6/chief nurse aide)

*“I thought of myself as I was many years ago and of myself as I am now. Now I feel more useful because I have experience of things already seen and experienced. It is a matter of experience that has been gained in the field”.*

(NH6/psychologist)

HCPs identified mostly practical education needs to sustain communication with FCs at the end of life. Topics included how to support FCs with strong emotions, finding the right words and manner to approach FCs, and personalizing communication. A few participants reported a desire for theoretical knowledge, including how to deal with different personality types they may experience when meeting FCs.

*“I’d like to take classes on how to engage with angry FCs. It is not easy to say the right words or have the right attitudes when someone is angry because of a feeling of helplessness. It is not easy to manage and contain such feeling of helplessness.”*

(NH5/nurse aide)

All participants believed that communication training should be offered to both clinical and administrative staff who work in NHs. The HCPs wanted this training to be practical and consistent with the context of their working environment. Among the training modalities perceived as most useful, they suggested case discussions based on real clinical scenarios that the multi-professional team had encountered. Face-to-face, small-group training was preferred even if some HCPs recognized the potential usefulness of online, asynchronous training with the opportunity to download hard copy resources. Practical tools available to them at the point of care were also requested. In addition, video and role play may be useful to facilitate dynamic learning. To reinforce and stabilize learning, HCPs highlighted the pivotal role of recurring, regular meetings of the multi-professional team over time.

*“I strongly believe in case-based discussions in team. We start from the specific case to reflect on how we behaved and if we could have behaved differently, also as a warning for the future.”*

(NH2/NH manager)

*“I remember a course I did, we were few and it was nice because it gave me the opportunity to confront with my colleagues and make a sort of exchange.”*

(NH5/nurse aide)

*“We recently did a course on communication in the form of role-plays. There was a trained actress on what she had to say. […] It gave me several ideas on how managing meetings with FCs, it gave me a mental outline to move. Obviously, every meeting is unique but a practical basis to start is useful.”*

(NH5/chief nurse aide)

## 4. Discussion

This qualitative study explored the challenges experienced by Italian HCPs in both initiating and continuing end-of-life conversations with FCs of NH residents during COVID-19 pandemic, to gain knowledge on what communication skills training might need to be prioritized. Our qualitative analysis identified four major themes, including (1) communicating with FCs over the overall disease trajectory, (2) managing challenging emotions and situations, (3) establishing a partnership between HCPs and FCs, and (4) addressing HCPs’ communication skills and training needs.

Our findings showed that HCPs perceived end-of-life conversations with FCs as a continuum over the overall disease trajectory, since the resident moves to the NH until death and beyond. Our interviewees reported a continuous adjustment of communication to address FCs’ needs, with increasingly emotional needs as the resident moves toward death, while clinical information needs progressively decrease. Consistent with the literature, our interviewees reported that transitioning into a NH is stressful for most FCs, who may feel guilty regarding the decision to admit their relative to a NH [35,36]. Then, when a resident’s condition deteriorates and death approaches, FCs need education about possible care options and support in navigating difficult decisions such as the withholding or withdrawal of intensive treatments, including artificial nutrition and intravenous hydration [37]. Moreover, our findings suggested that end-of-life conversations play a critical role after the residents’ death to transition their FCs into the bereavement phase. Communication of deterioration was experienced as one of the most challenging communication scenarios. This explains poor or delayed communication [12,38], with a third of patients dying without any conversations with the physician [39], and a further third having such conversations in the last month of life [40]. Our interviewees agreed that professionalism, caring attitudes, and being well-informed about the resident’ medical history was crucial for successful end-of-life conversations, particularly in conditions of visitation restrictions, such as during the COVID-19 pandemic [41]. They largely relied on ICT-based communication and increased the frequency of remote contacts to stay close to FCs and satisfy their information and emotional needs. The literature supports this strategy, showing that FCs highly valued HCPs’ competence and their efforts to maintain a therapeutic relationship between staff and their relative by employing a variety of means of communication [42,43].

Our data showed that HCPs had to overcome several challenging situations throughout a resident’s time in the NH, including FCs’ denial of their relative’s worsening, prognostic discordance, and mismatched care expectations. Experts recommend that clarifying the FCs perception regarding the illness is among the main strategies prior to a successful prognostic conversation, in addition to responding to emotion, exploring values, and making recommendations for medical treatments that fit FCs’ values [44,45,46]. However, discussing prognosis with a FC whose relative suffers from dementia or multiple chronic conditions may be hard for HCPs since the clinical course is difficult to predict, while other terminal diagnoses, such as cancer, seem to be more clearly understood [16]. Moreover, the content as well as the delivery and timing of conversation need to be tailored to the FCs’ desire for information and levels of understanding [30]. Our HCPs struggled to balance the need for being honest while building trust and avoiding false hope. They had to tailor the level of language used relative to the FCs’ acceptance of dementia as a cause of death. During the pandemic, it became even harder to prepare FCs for death since they could not regularly check their relative’s general health condition. The interviewees recognized the importance in helping FCs recognize a poor prognosis following admission, thus avoiding requests of nonproportionate treatments [47]. Moreover, they highlighted the need to employ a clear communication to promptly scale down FCs’ unrealistic expectations. When such challenging situations are not adequately pro-actively managed, FCs often experience frustration or distress. This may result in FCs being unprepared for clinical deterioration or even imminent death, limiting shared decision-making, and increasing the risk of more aggressive treatments [48].

HCPs also had to support FCs through strong emotions (varying from anxiety, guilt, and uncertainty at admission to anger and suffering as death approaches), and the HCPs may also experience complex feelings such as reliving painful personal experience, concerns about FCs’ reactions when providing bad news, and sadness when witnessing residents who died alone. The literature has largely addressed FCs’ emotional needs relating to end-of-life conversations and recognized HCPs’ response to FCs’ emotions of fear, sadness, and grief as a critical support process for sustaining FCs through the process of loss and through the transition into bereavement [44]. Instead, HCPs’ experience of end-of-life conversations has been overlooked [48,49]. We found that enabling interactions between FCs and their relative may be challenging for HCPs, particularly when employing ICT-based modalities at the end of life. The mediator role in these circumstances sometimes made HCPs suffer and negatively affected their well-being. Conversing with colleagues in structured, peer-facilitated, informal groups was an opportunity to recognize the impact of the work on them, normalize emotions, and learn coping strategies [50,51]. Our findings point out that beyond communication skills training, any intervention should also provide HCPs with emotional and social support in an environment that allows time for reflective practice both at the individual and at the care team level.

Fulfilling FCs’ needs and trusting relationships with the NH team created a partnership between FCs and HCPs [52]. HCPs were more comfortable to sustain end-of-life conversations when they had face-to-face meetings and had established relationships with the patient and FCs. They found it difficult to develop relationships when residents were admitted after the closing of NHs, thus suggesting the irreplaceable role of in-person communication in promoting trust. Consistent with previous works exploring communication between HPCs and FCs of persons approaching end of life [30], we found that involving FCs in shared decisions and exploring their preferences for end-of-life care facilitated a collaborative process in making decisions for end-of-life care. Highlighting deterioration and ongoing problems usually provided FCs with enough evidence that their relative was reaching end of life and further active treatments would be nonbeneficial, thus leading to a shared comfort-oriented approach decision. However, it was often not easy to elicit FCs’ care preferences since most FCs still experienced death as a taboo [53]. Moreover, as previously highlighted [54,55], FCs’ care preferences were rarely static and were influenced by their relative’s health status in addition to a sense of obligation and responsibilities associated with the social role expectations; FCs were often driven by a filial duty to leave nothing undone, which was paired with the misbelief that opting for a comfort-oriented approach meant abandonment of the relative. Lack of time, poor or fragmented communication among HCPs, and further increase in the known nursing staff turnover and shortages during the pandemic after extra nurses were called into hospitals to deal with the care pressure from COVID-19 patients were confirmed to be barriers to initiating and sustaining end-of-life conversations [16]. Consistent with previous authors [15,30], our interviewees argued the role of different HCPs in end-of-life conversations and complained of poor role clarity, which may be responsible for conflicting and confusing information. This does not mean that end-of-life conversations should be led by senior or high-rank HCPs by default, but rather it highlights the need to define clear role boundaries about which information each HCP should provide based on the field of competence and when FCs’ questions should be handed over to a colleague. In Italy, the long-term sector is a regional competence and largely varies across regional jurisdictions in staff-to-residents ratio and organization of internal processes, including end-of-life communication. However, regardless of the model of care, end-of-life communication has been described as a “hot potato,” whereby everyone and no one is taking ownership [11]. Within the Italian landscape, there is current debate concerning the introduction of a geriatrician with adequate training in palliative care in each NH [56]. Having an internal physician or identifying a single reference figure for communication with FCs may facilitate the coordination of a team-based approach for end-of-life communication, which is recognized as the best approach from both an economic and a quality effectiveness standpoint [57].

HCPs also suggested that communication training should be tailored to their scope of practice. The literature supports a team-based model for successful conversations by dividing responsibilities among team members and alleviating pressure [1]. Physicians were often seen as HCPs responsible for conducting prognostic and decision-making conversations, while nurses often promoted FCs’ understanding by translating what physicians had said into jargon-free information and bringing together information from different sources [30].

We found that most HCPs gained communication skills in the field, thus confirming that experience is positively associated with comfort in communicating about end-of-life care [31]. Despite this, they often felt ill-equipped for end-of-life conversations, thus laying bare a lack of training and education [20,58] notwithstanding that this has been identified as a core aspect to provide optimal comfort-oriented care at the end of life [9]. Such inadequate education and training of NH staff is also suggested by the poor control of distressing symptoms at the end of life [59,60] and FCs dissatisfaction with care [61]. This leads to the urgent call for educational interventions that have the potential to improve end-of-life care in NHs, including interventions aimed to enhance communication skills using various modalities [62]. Our interviewees would largely welcome communication training initiatives, consistent with an international survey of palliative care experts that found 83% agreement for the need for more evidence-based guidance on strategies for end-of-life conversation with FCs [2]. Particularly, they judged practical training programs such as team-based discussions of real cases as the most adequate to develop communication skills while reflecting about their experience, since their education needs were mostly practical in nature. Unfortunately, current evidence about educational interventions aimed at providing HCPs with training in end-of-life conversations skills is limited by poor reporting and weak methodology [63]. Evidence that end-of-life conversations training improved self-efficacy, knowledge and communication score was consistent, but the low-quality data were insufficient to drive conclusions at the patient level [64]. Therefore, further methodologically sound interventions aimed at improving HCPs’ conversation skills are needed. Investment in HCPs training is an expression of a culture change that is ongoing and needs to be sustained also for the potential to improve NH quality of care [65]. All NHs adhering to the project had written procedures to welcome residents and their FCs at admission, five out of six provided HCPs with guidance on communication of deterioration, but only three had bereavement procedures and two pain management procedures for cognitively impaired residents. NH leadership is well-positioned to sustain a conducive environment to promote change [66].

### Limitations

This study involved a purposeful sample of NHs that adhered to the project on a voluntarily basis and were thus more likely to have sensitivity towards end-of-life conversations. Therefore, this may have prevented further challenges from emerging and provided a biased positive picture of challenges experienced by HCPs in end-of-life conversations during pandemic. Secondly, this study was performed in Northwest Italian NHs, and none had internal physicians who may be important figures of contact for end-of-life communication; this may limit the transferability of our findings to other regional jurisdictions and health systems. Thirdly, as suggested by the excerpts reported in the article, the perspective of educators and nurse aides was poorly represented. This may be an expression of the limited involvement of these HCPs in end-of-life conversations in Italian NH settings, where a team-based approach to end-of-life conversations is still in its infancy and they are mostly led by NH managers who are informed by the care staff when a resident’s clinical conditions deteriorate. However, HCPs with a wide range in years of working experience and different working profiles have been recruited, thus offering multiple facets of challenges in conversations with FCs at the end of life.

## 5. Conclusions

HCPs reported that conversations with FCs of NH residents should be a continuum over the overall disease trajectory and tailored to their specific and changing information and supportive needs. HCPs had to face multiple challenging situations, which varied across the entire stay at the NH, including poor awareness or denial of a relative’s worsening condition, unrealistic expectations for care, and exploration of care preferences in a death-averse society with a Catholic tradition where truth must be told without destroying hope. Moreover, they had to manage personal emotions and address FCs’ complex feelings such as anxiety, guilt, uncertainty, fear, anger, or suffering, with remote modalities creating additional barriers. Anxiety and guilt were predominant at admission and gradually replaced by anger and suffering as death approached, thus requiring phase-tailored approaches. Moreover, the COVID-19 pandemic increased FCs’ aggression, frustration, distrust, and uncertainty possibly due to visitation restrictions, which HCPs had to overcome by setting up a set of strategies, including adoption of an active-listening approach and supportive communication as well as acknowledgement of FCs’ emotions. Fulfilling FCs’ needs and trusting relationships promoted the establishment of a partnership with HCPs, which was essential for true shared decision-making, despite the threat of staff shortages, turnover, and lack of time.

Our findings highlighted mostly practical communication needs, which HCPs perceived to be better addressed by equally practical communication skills training, which should be open to all HCPs, directed by their scope of practice, and employ several educational methods—case discussions, video, and role play, provided in multiple sessions, face-to-face, and in a small group to reinforce learning, if possible.

## Figures and Tables

**Table 1:** Characteristics of nursing homes adhering to the study (*n* = 6).

Characteristics	N
**STRUCTURE VARIABLES**	
**Private/public NH**	4/2
**Number of beds available**, mean (range)	81 (37–122)
≤60	2
61–119	3
≥120	1
**Number of beds for functionally independent residents**, mean (range)	12.4 (0–40)
**Number of beds for functionally dependent residents**, mean (range)	57.7 (14–122)
**Alzheimer unit**	2
**Facilities with beds of palliative care continuity**	1
**Staffing**, full-time equivalent, mean (SD)	
Nurse aide	39.0 (22.0)
Nurse	5.7 (2.8)
Physiotherapist	1.02 (0.21)
Physician	0.7 (0.5)
Educator	0.66 (0.51)
Psychologist	0.5 (0.2)
**PROCESS VARIABLES**	
**Presence of written procedures on**	
how to welcome residents and their families at admission	6
how to communicate resident’s clinical deterioration and/or end-of-life conditions	5
pain management in cognitively competent residents	6
pain management in cognitively impaired residents	2
bereavement management	3
**Meetings with family caregivers**	
when a resident’s condition worsens	6
to explore family caregivers’ preferences for end-of-life care	5
to explore their spiritual needs	4
to present the opportunity to activate the palliative care service in the 6 months before the study start	4
to present the opportunity of hospice referral in the 6 months before the study start	-
**Meetings with residents**	
to explore their preferences for end-of-life care	4
to explore their spiritual needs	4
**Documentation of residents’ and/or family caregivers preferences for end-of-life care**	4
**Availability of a priest on regular basis**	6
**Availability to reach other spiritual guides on request**	4
**Figures involved in answering family caregivers’ concerns during the facility selection**	
NH director	6
Chief medical officer	6
Nurse	6
Administrative staff	2
**Figures involved in updating the care plan**	
Chief medical officer	6
Nurse	6
Family caregivers	6
Psychologist	2
Nurse aide	2
Physiotherapist	1
**Figures involved in communicating a resident’s clinical deterioration to family caregivers**	
Chief medical officer	4
Nurse	3
NH director	2
**OUTCOME VARIABLES**	
**Referral of residents to palliative care services in the 6 months before study start**	1
**Extra activities**	
Pet therapy	4
Music therapy	3
Occupational therapy	3
Bio-dance	2
Board games	2
Others ^a^	3

Abbreviations. NH, Nursing home. ^a^ Doll therapy (*n* = 1), garden therapy (*n* = 1), and projects with kindergartens (*n* = 1).

**Table 2:** Participants’ characteristics.

Healthcare Professionals (*n* = 21)	N
**Female gender**	17
**Age**, years, mean (range)	50 (25–73)
**Education**	
Junior high school licence	2
Bachelor degree	10
Master degree	9
**Professional profile**	
Nursing home manager	4
Chief nurse	4
Chief medical officer	3
Nurse	3
Psychologist	3
Educator	2
Chief nurse aide	1
Nurse aide	1
**Overall working experience**, years, mean (range)	16 (1–50)
**Working experience in nursing home**, years, mean (range)	8.5 (0.5–25)
**Employment**	
Permanent full-time	14
Freelance Permanent part-time	6
Permanent part-time	1

**Table 3:** Themes and categories emerged from interviews with healthcare professionals.

Themes	Communicating with Family Caregivers over the Overall Disease Trajectory	Managing Challenging Emotions and Situations	Establishing a Partnership between Healthcare Professionals and Family Caregivers	Addressing Healthcare Professionals’ Communication Skills and Training Needs
**Categories**	Supportive communication Traditional communication Remote communication Healthcare professionals’ attitudes and professionalism Admission phase Approaching and at end-of-life phase After death phase	Managing family caregivers’ denial of the resident’s worsening condition Managing prognostic discordance Managing family caregivers’ expectations Managing healthcare professionals’ uncertainty about prognosis Managing family caregivers’ complex and turbulent emotions Managing healthcare professionals’ complex and turbolent emotions Overcoming challenges	Fulfilling family caregivers’ information and supportive needs Establishing trusting relationships Sharing decisions with family caregivers and among healthcare professionals Exploring family caregivers’ preferences for end-of-life care Healthcare professionals’ shortage and burden Time constraints	Source of communication skills Healthcare professionals’ education needs to sustain communication with family caregivers Preferred training to gain communication skills

## Data Availability

Data are contained within the article or Supplementary Material.

## Supplementary Materials

The following supporting information can be downloaded at https://www.mdpi.com/article/10.3390/ijerph19052504/s1, Table S1: Themes, categories, and codes.

## References

[1] Pfeifer M., Head B.A. (2018). Which critical communication skills are essential for interdisciplinary end-of-life discussions?. AMA J. Ethics.

[2] Raijmakers N.J., van Zuylen L., Costantini M., Caraceni A., Clark J.B., De Simone G., Lundquist G., Voltz R., Ellershaw J.E., van der Heide A. (2012). Issues and needs in end-of-life decision making: An international modified Delphi study. Palliat. Med..

[3] Gonella S., Basso I., Dimonte V., Martin B., Berchialla P., Campagna S., Di Giulio P. (2019). Association Between End-of-life conversations in nursing homes and end-of-life care outcomes: A systematic review and meta-analysis. J. Am. Med. Dir. Assoc..

[4] Street R.L., Makoul G., Arora N.K., Epstein R.M. (2009). How does communication heal? Pathways linking clinician–patient communication to health outcomes. Patient Educ. Couns..

[5] Sanders J.J., Curtis J.R., Tulsky J.A. (2018). Achieving goal-concordant care: A conceptual model and approach to measuring serious illness communication and its impact. J. Palliat. Med..

[6] Teno J.M., Clarridge B.R., Casey V., Welch L.C., Wetle T., Shield R., Mor V. (2004). Family perspectives on end-of-life care at the last place of care. JAMA.

[7] Sinuff T., Dodek P., You J.J., Barwich D., Tayler C., Downar J., Hartwick M., Frank C., Stelfox H.T., Heyland D.K. (2015). Improving end-of-life communication and decision making: The development of a conceptual framework and quality indicators. J. Pain Symptom Manag..

[8] Dy S.M., Kiley K.B., Ast K., Lupu D., Norton S.A., McMillan S.C., Herr K., Rotella J.D., Casarett D.J. (2015). Measuring what matters: Top-ranked quality indicators for hospice and palliative care from the American Academy of Hospice and Palliative Medicine and Hospice and Palliative Nurses Association. J. Pain Symptom Manag..

[9] van der Steen J.T., Radbruch L., Hertogh C.M., de Boer M.E., Hughes J.C., Larkin P., Francke A.L., Jünger S., Gove D., Firth P. (2014). White paper defining optimal palliative care in older people with dementia: A Delphi study and recommendations from the European Association for Palliative Care. Palliat. Med..

[10] Bowman P., Slusser K., Allen D. (2018). Collaborative practice model: Improving the delivery of bad news. Clin. J. Oncol. Nurs..

[11] Harrison Dening K. (2016). Advance Care Planning in Dementia.

[12] Gjerberg E., Lillemoen L., Førde R., Pedersen R. (2015). End-of-life care communications and shared decision-making in Norwegian nursing homes—Experiences and perspectives of patients and relatives. BMC Geriatr..

[13] Hancock K., Clayton J.M., Parker S.M., Wal der S., Butow P.N., Carrick S., Currow D., Ghersi D., Glare P., Hagerty R. (2007). Truth-telling in discussing prognosis in advanced life-limiting illnesses: A systematic review. Palliat. Med..

[14] Almack K., Cox K., Moghaddam N., Pollock K., Seymour J. (2012). After you: Conversations between patients and healthcare professionals in planning for end of life care. BMC Palliat. Care.

[15] Bergenholtz H., Timm H.U., Missel M. (2019). Talking about end of life in general palliative care—What’s going on? A qualitative study on end-of-life conversations in an acute care hospital in Denmark. BMC Palliat. Care.

[16] De Vleminck A., Pardon K., Beernaert K., Deschepper R., Houttekier D., Van Audenhove C., Deliens L., Stichele R.V. (2014). Barriers to advance care planning in cancer, heart failure and dementia patients: A focus group study on general practitioners’ views and experiences. PLoS ONE.

[17] Schonfeld T.L., Stevens E.A., Lampman M.A., Lyons W.L. (2012). Assessing challenges in end-of-life conversations with elderly patients with multiple morbidities. Am. J. Hosp. Palliat. Med..

[18] Gonella S., Basso I., Clari M., Dimonte V., Di Giulio P. (2021). A qualitative study of nurses’ perspective about the impact of end-of-life communication on the goal of end-of-life care in nursing home. Scand. J. Caring Sci..

[19] Pino M., Parry R., Land V., Faull C., Feathers L., Seymour J. (2016). Engaging terminally ill patients in end of life talk: How experienced palliative medicine doctors navigate the dilemma of promoting discussions about dying. PLoS ONE.

[20] Travers A., Taylor V. (2016). What are the barriers to initiating end-of-life conversations with patients in the last year of life?. Int. J. Palliat. Nurs..

[21] Rubinelli S., Myers K., Rosenbaum M., Davis D. (2020). Implications of the current COVID-19 pandemic for communication in healthcare. Patient Educ. Couns..

[22] Mitchinson L., Dowrick A., Buck C., Hoernke K., Martin S., Vanderslott S., Robinson H., Rankl F., Manby L., Lewis-Jackson S. (2021). Missing the human connection: A rapid appraisal of healthcare workers’ perceptions and experiences of providing palliative care during the COVID-19 pandemic. Palliat. Med..

[23] Hanna J.R., Rapa E., Dalton L.J., Hughes R., Quarmby L.M., McGlinchey T., Donnellan W.J., Bennett K.M., Mayland C.R., Mason S.R. (2021). Health and social care professionals’ experiences of providing end of life care during the COVID-19 pandemic: A qualitative study. Palliat. Med..

[24] Batra K., Singh T.P., Sharma M., Batra R., Schvaneveldt N. (2020). Investigating the psychological impact of COVID-19 among healthcare workers: A meta-analysis. Int. J. Environ. Res. Public Health.

[25] Smaling H.J.A., Tilburgs B., Achterberg W.P., Visser M. (2022). The impact of social distancing due to the COVID-19 pandemic on people with dementia, family carers and healthcare professionals: A qualitative study. Int. J. Environ. Res. Public Health.

[26] Hado E., Feinberg L.F. (2020). Amid the COVID-19 pandemic, meaningful communication between family caregivers and residents of long-term care facilities is imperative. J. Aging Soc. Policy.

[27] Sandelowski M. (2000). Whatever happened to qualitative description?. Res. Nurs. Health.

[28] Tong A., Sainsbury P., Craig J. (2007). Consolidated criteria for reporting qualitative research (COREQ): A 32-item checklist for interviews and focus groups. Int. J. Qual. Health Care.

[29] Guest G., Bunce A., Johnson L. (2006). How many interviews are enough? An experiment with data saturation and variability. Field Methods.

[30] Anderson R.J., Bloch S., Armstrong M., Stone P., Low J. (2019). Communication between healthcare professionals and relatives of patients approaching the end-of-life: A systematic review of qualitative evidence. Palliat. Med..

[31] Moir C., Roberts R., Martz K., Perry J., Tivis L.J. (2015). Communicating with patients and their families about palliative and end-of-life care: Comfort and educational needs of nurses. Int. J. Palliat. Nurs..

[32] Braun V., Clarke V. (2006). Using thematic analysis in psychology. Qual. Res. Psychol..

[33] Morse J.M. (2015). Critical analysis of strategies for determining rigor in qualitative inquiry. Qual. Health Res..

[34] Holloway I., Galvin K. (2016). Qualitative Research in Nursing and Healthcare.

[35] Konietzny C., Kaasalainen S., Haas V.D.-B., Merla C., Te A., Di Sante E., Kalfleish L., Hadjistavropoulos T. (2018). Muscled by the system: Informal caregivers’ experiences of transitioning an older adult into long-term care. Can. J. Aging/La Rev. Can. Vieil..

[36] Merla C., Wickson-Griffiths A., Kaasalainen S., Bello-Haas V.D., Banfield L., Hadjistavropoulos T., Di Sante E. (2018). Perspective of family members of transitions to alternative levels of care in Anglo-Saxon countries. Curr. Gerontol. Geriatr. Res..

[37] mySupport Study Scaling up the Family Carer Decision Support Intervention: A Transnational Effectiveness-Implementation Evaluation. 2019–2022. https://mysupportstudy.eu.

[38] Toles M., Song M.-K., Lin F.-C., Hanson L.C. (2018). Perceptions of family decision-makers of nursing home residents with advanced dementia regarding the quality of communication around end-of-life care. J. Am. Med. Dir. Assoc..

[39] Melin-Johansson C., Sveen J., Lövgren M., Udo C. (2021). A third of dying patients do not have end-of-life discussions with a physician: A nationwide registry study. Palliat. Support. Care.

[40] Reinke L.F., Feemster L.C., McDowell J., Gunnink E., Tartaglione E.V., Udris E., Curtis J.R., Au D.H. (2017). The long term impact of an end-of-life communication intervention among veterans with COPD. Heart Lung.

[41] Miralles O., Sanchez-Rodriguez D., Marco E., Annweiler C., Baztan A., Betancor É., Cambra A., Cesari M., Fontecha B.J., Gąsowski J. (2020). Unmet needs, health policies, and actions during the COVID-19 pandemic: A report from six European countries. Eur. Geriatr. Med..

[42] Wammes J.D., Kolk M.D., van den Besselaar Md J.H., MacNeil-Vroomen Ph D.J., Buurman-van Es Rn B.M., van Rijn Ph D.M. (2020). Evaluating perspectives of relatives of nursing home residents on the nursing home visiting restrictions during the COVID-19 crisis: A Dutch cross-sectional survey study. J. Am. Med. Dir. Assoc..

[43] Zmora R., Statz T.L., Birkeland R.W., McCarron H.R., Finlay J.M., Rosebush C.E., Gaugler J.E. (2021). Transitioning to long-term care: Family caregiver experiences of dementia, communities, and counseling. J. Aging Health.

[44] Childers J.W., Back A.L., Tulsky J.A., Arnold R.M. (2017). REMAP: A framework for goals of care conversations. J. Oncol. Pract..

[45] Haley E.M., Meisel D., Gitelman Y., Dingfield L., Casarett D.J., O’Connor N.R. (2017). Electronic goals of care alerts: An innovative strategy to promote primary palliative care. J. Pain Symptom Manag..

[46] VitalTalk VitalTalk Makes Communication Skills for Serious Illness Learnable. https://www.vitaltalk.org/.

[47] Valdimarsdóttir U., Helgason Ásgeir R., Fürst C.-J., Adolfsson J., Steineck G. (2004). Awareness of husband’s impending death from cancer and long-term anxiety in widowhood: A nationwide follow-up. Palliat. Med..

[48] Cagle J.G., Unroe K.T., Bunting M., Bernard B.L., Miller S.C. (2017). Caring for dying patients in the nursing home: Voices from frontline nursing home staff. J. Pain Symptom Manag..

[49] Brighton L.J., Selman L.E., Bristowe K., Edwards B., Koffman J., Evans C.J. (2019). Emotional labour in palliative and end-of-life care communication: A qualitative study with generalist palliative care providers. Patient Educ. Couns..

[50] Borghi L., Meyer E.C., Vegni E., Oteri R., Almagioni P., Lamiani G. (2021). Twelve years of the Italian Program to Enhance Relational and Communication Skills (PERCS). Int. J. Environ. Res. Public Health.

[51] James H., Crawford G.B. (2021). Healthcare interpreters and difficult conversations: A survey. BMJ Support. Palliat. Care.

[52] Gonella S., Basso I., De Marinis M.G., Campagna S., Di Giulio P. (2019). Good end-of-life care in nursing home according to the family carers’ perspective: A systematic review of qualitative findings. Palliat. Med..

[53] Toscani F., Farsides C. (2006). Deception, catholicism, and hope: Understanding problems in the communication of unfavorable prognoses in traditionally-catholic countries. Am. J. Bioeth..

[54] Bern-Klug M. (2014). A conceptual model of family surrogate end-of-life decision-making process in the nursing home setting: Goals of care as guiding stars. J. Soc. Work End-of-Life Palliat. Care.

[55] Morrison R.S., Meier D.E., Arnold R.M. (2021). What’s wrong with advance care planning?. JAMA.

[56] Anziani e pandemia Geriatri: “Rsa, Piano Vaccinale Battaglia Vinta. Ma serve un Geriatra in Ogni Struttura”. https://www.quotidianosanita.it/lavoro-e-professioni/articolo.php?articolo_id=101864.

[57] Dixon J., Knapp M. (2018). Whose job? The staffing of advance care planning support in twelve international healthcare organizations: A qualitative interview study. BMC Palliat. Care.

[58] Evenblij K., Koppel M.T., Smets T., Widdershoven G.A.M., Onwuteaka-Philipsen B.D., Pasman H.R.W. (2019). Are care staff equipped for end-of-life communication? A cross-sectional study in long-term care facilities to identify determinants of self-efficacy. BMC Palliat. Care.

[59] Mitchell S.L., Teno J.M., Kiely D.K., Shaffer M.L., Jones R., Prigerson H.G., Volicer L., Givens J.L., Hamel M.B. (2009). The clinical course of advanced dementia. N. Engl. J. Med..

[60] Smedbäck J., Öhlén J., Årestedt K., Alvariza A., Fürst C.-J., Håkanson C. (2017). Palliative care during the final week of life of older people in nursing homes: A register-based study. Palliat. Support. Care.

[61] Thompson G.N., McClement S., Menec V.H., Chochinov H.M. (2012). Understanding bereaved family members’ dissatisfaction with end-of-life care in nursing homes. J. Gerontol. Nurs..

[62] Anstey S., Powell T., Coles B., Hale R., Gould D. (2016). Education and training to enhance end-of-life care for nursing home staff: A systematic literature review. BMJ Support. Palliat. Care.

[63] Brighton L.J., Koffman J., Hawkins A., McDonald C., O’Brien S., Robinson V., Khan S.A., George R., Higginson I.J., Selman L.E. (2017). A systematic review of end-of-life care communication skills training for generalist palliative care providers: Research quality and reporting guidance. J. Pain Symptom Manag..

[64] Chung H.-O., Oczkowski S.J.W., Hanvey L., Mbuagbaw L., You J.J. (2016). Educational interventions to train healthcare professionals in end-of-life communication: A systematic review and meta-analysis. BMC Med. Educ..

[65] Grabowski D.C., O’malley A.J., Afendulis C.C., Caudry D.J., Elliot A., Zimmerman S. (2014). Culture change and nursing home quality of care. Gerontologist.

[66] Nilsen P., Wallerstedt B., Behm L., Ahlström G. (2018). Towards evidence-based palliative care in nursing homes in Sweden: A qualitative study informed by the organizational readiness to change theory. Implement. Sci..

