# Supplementary material for: Challenges Experienced by Italian Nursing Home Staff in End-of-Life Conversations with Family Caregivers during COVID-19 Pandemic: A Qualitative Descriptive Study
Source: Int J Environ Res Public Health. 2022 Feb 22;19(5):2504. doi: 10.3390/ijerph19052504 (PMC8909457; doi:10.3390/ijerph19052504)
Supplement: Supplementary file 1 [file ijerph-19-02504-s001.zip › ijerph-1589912-supplementary.pdf]

Table S1: Themes, categories, and codes

Themes	Categories	Codes
Communicating with family caregivers over the overall disease trajectory	Supportive communication	Truthful<br>Continuous<br>Clear<br>Providing emotional support<br>Gradual<br>Tailored<br>Provided by an informed healthcare professional
	Healthcare professionals' attitudes and professionalism	Professionalism<br>Empathy<br>Availability<br>Kindness<br>Sensitivity<br>Humanity<br>Patience<br>Active listening and silence<br>Suspension of judgment
	Traditional communication	Preferred over ICT-based communication<br>Preferred at residens' admission<br>Preferred when residents' clinical conditions deteriorate<br>Preferred at the end of life<br>Support to family caregivers allowed through non-verbal communication<br>Understanding of family caregivers' non-verbal communication allowed
	Remote communication	By telephone<br>By video-calls<br>By email<br>By text messages<br>Complementary to in-person communication in selected cases<br>Lack of body language<br>Possible misunderstandings<br>Risk to "miss pieces"<br>Difficulty to provide emotional support<br>Impossibility to check understanding of information
	Admission phase	Mutual acquaintace between healthcare professionals and family caregivers<br>Need to match resident's needs and care provided<br>Phase more challenging for family caregivers than their relative<br>Supporting family caregivers in the decision to transfer their relative to the nursing home

	Approaching and at end-of-life phase	Providing information about the possibility of impending death<br>Providing information about life expectancy<br>Providing information about death and dying<br>Greater difficulty in initiating than sustaining end-of-life communication<br>Some of the most emotionally challenging communications<br>Easier communication when strong relationships have been established over time
	After death phase	After death communication<br>Increased challenges in communication when family caregivers could not be present at the bedsite<br>Family caregivers' satisfaction with communication in presence of trusting relationships
Managing challenging emotions and situations	Managing family caregivers' denial of the resident's worsening condition	Family caregivers' denial of residents' clinical deterioration<br>Family caregivers' denial of cognitive deterioration in residents with dementia<br>Family caregivers' denial of dementia as a cause of death
	Managing prognostic discordance	Trusting relationships threatened by family caregivers' unawareness<br>Family caregivers' request for treatments judged non-proportionate for the resident (e.g., oral feeding, walking)<br>Family caregivers' surprise at the end of life<br>Increased family caregivers' unawareness during the pandemic
	Managing family caregivers' expectations	Family caregivers' unrealistic expectations<br>Scaling back family caregivers' expectations
	Managing healthcare professionals' uncertainty about prognosis	Not knowing what to answer<br>No certainty about the amount of time left
	Managing family caregivers' complex and	Uncertainty during the institutionalization phase<br>Anxiety during the institutionalization phase<br>Guilt during the institutionalization phase<br>Fear of relative's post-admission worsening due to changed routine<br>Anger

	turbolent emotions	Suffering at the end of the relative' s life<br>Increased aggression during the pandemic<br>Increased distrust during the pandemic<br>Increased dissatisfaction during the pandemic<br>Uncertainty related to in-person presence not allowed during the pandemic<br>Fear of their relative dying alone during the pandemic
	Managing healthcare professionals' complex and turbulent emotions	Reliving painful personal experiences<br>Sadness for residents dying without their relatives at the bedsite during the pandemic<br>Fear of family caregivers' reactions after bad news<br>Feeling heartrending when witnessing remote communication between family caregivers and their actively dying relative
	Overcoming challenges	Explicitly acknowledging family caregivers emotions<br>Helping family caregivers to acknowledge their relative's changed clinical conditions<br>Listening to rather than hearing<br>Focusing on the content of communication<br>Transferring responsibility of communication to a superior<br>Multi-professional meetings<br>Defining which information to be given based on the professional role
Establishing a partnership between healthcare professionals and family caregivers	Fulfilling family caregivers' information and supportive needs	Understanding family caregivers' needs<br>Need for reassurance<br>Need for emotional support<br>Need for detailed clinical information<br>Need for increased emotional support during the pandemic<br>Need to stay in touch with the relative (e.g., through in-person visits, window visits, outdoor visits, video-calls)
	Establishing trusting relationships	Pivotal importance of trust<br>Healthcare professionals' commitment required<br>Facilitated by matched care goals<br>Promoted by in-person communication<br>Difficult to be established with family caregivers of residents admitted during the pandemic<br>Difficult to be established with family intrusive and critical family caregivers<br>Difficult to be established with family units characterised by internal conflicts
	Sharing decisions with family caregivers and among	Hospitalization at the end of life<br>Place of death<br>Physical restraints for cognitive impaired residents<br>Shared comfort-oriented approach with family caregivers<br>Decisions shared after communication of deterioration<br>Difficulty to support family caregivers' desire to pursue curative-oriented care

	healthcare professionals	Disagreement among healthcare providers (e.g., hospitalization, specialist medical visits, end-of-life support)
	Exploring family caregivers' preferences for end-of-life care	Lack of culture of advance directives<br>Death as a taboo<br>Filial duty to leave nothing undone<br>Family caregivers' belief to abandon their relative if a comfort-oriented approach was chosen<br>Use of indirect questions to explore family caregivers' preferences for care at the end of life<br>Changing preferences from admission until end of life
	Healthcare professionals' shortage and burden	Fragmented communication with and within the care team<br>Turnover<br>Staff shortages
	Time constraints	Lack of time to communicate with family caregivers<br>To be in a rush
Addressing healthcare professionals' communication skills and training needs	Source of communication skills	Communication skills as hard-wired/innate abilities<br>Communication skills gained through working experience<br>Communication skills as result of personal experiences of caring for relatives<br>Communication training programmes during education<br>Absent or poor training offered by the nursing home
	Healthcare professionals' education needs to sustain communication with family caregivers	Sustaining communication at the end of life<br>Personalizing communication<br>Finding the right words<br>Finding the right manner to approach family caregivers<br>Managing one's emotions<br>Supporting family caregivers with strong emotions<br>Theoretical knowledge
	Preferred training to gain communication skills	Open to both clinical and administrative staff<br>Tailored on the profile<br>Case discussions based on real clinical scenarios encountered by the multi-professional team<br>In-presence, small group training<br>Online, asynchronous training<br>Printed, synthetic material<br>Practical tools available during conversation<br>Video<br>Role play<br>Recurring, regular meetings of the care team over time
